# Clinical and Histological Features of Ovarian Hypoplasia/Dysgenesis in Alpacas

**DOI:** 10.3389/fvets.2022.837684

**Published:** 2022-03-25

**Authors:** Eduardo Arroyo, Cristian Patiño, Michela Ciccarelli, Terje Raudsepp, Alan Conley, Ahmed Tibary

**Affiliations:** ^1^Comparative Theriogenology Section, Department of Veterinary Clinical Sciences, College of Veterinary Medicine, Pullman, WA, United States; ^2^Center for Reproductive Biology, Washington State University, Pullman, WA, United States; ^3^Department of Veterinary Integrative Biosciences, Texas A&M University, College Station, TX, United States; ^4^Department of Population Health and Reproduction, School of Veterinary Medicine, University of California, Davis, Davis, CA, United States

**Keywords:** *Vicugna pacos*, infertility, histopathology, cytogenetics, ovary, laparoscopy, AMH

## Abstract

Alpacas have a high incidence of congenital reproductive tract abnormalities, including ovarian hypoplasia/dysgenesis. Diagnosis of this condition is often challenging. The present study describes the clinical, ultrasonographic, and histologic features of ovarian hypoplasia/dysgenesis syndrome in 5 female alpacas. Additionally, serum AMH levels were compared between female alpacas diagnosed with ovarian hypoplasia/dysgenesis and a group of reproductively sound females (*n* = 11). The syndrome was suspected based on the presence of an infantile uterus and lack of ovaries by ultrasonography and laparoscopy. All females had normal female karyotype (*n* = 74 XX), but one presented a minute chromosome. The ovaries from these cases showed 3 main histological classifications: hypoplasia (*n* = 2), dysgenesis (*n* = 2), and dysplasia (*n* = 1). Serum AMH levels in affected females were significantly lower (*P* < 0.05) than those of reproductively sound control females. In conclusion, Serum AMH level may be helpful in the rapid diagnosis of ovarian hypoplasia/dysgenesis syndrome in alpacas. Furthermore, this syndrome in alpacas presents a variety of histological features. Different mechanisms may be involved in the derangement of ovarian differentiation. Further studies are needed to elucidate the causes of the syndrome.

## Introduction

Alpacas and llamas are important production animals in several South American countries (1). Alpacas were introduced into the USA, Australia, New Zealand, and several European countries recently as companion and fiber production animals. In these countries, the limited animal number and high sentimental and monetary investment have increased the emphasis on individual animal medicine. The inability to generate offspring is one of the most common reasons for examining alpacas in veterinary practice. Detailed protocols for investigating infertility were developed over the years (1).

In maiden female alpacas, congenital reproductive disorders are of particular interest because of the legal and welfare issues. Their diagnosis often causes a significant challenge to the veterinary practitioner. The most comprehensive description of congenital disorders of the reproductive system in alpacas was published by Sumar on abattoir specimens (2, 3). Ovarian hypoplasia was reported in 16.8% of 155 infertile alpacas following postmortem examination of the reproductive tract (3). Ovarian hypoplasia in alpacas and llamas has been associated with several types of chromosomal abnormalities (XXX, XO, XX/XY) and the presence of the *minute* chromosome (4–7). However, a significant proportion of animals diagnosed with ovarian hypoplasia have normal karyotype (1, 4).

The absence of ovarian follicular activity has been investigated in several species and may present in various histological features (i.e., hypoplasia, low germ cell resistance, and dysgenesis) (8). Although some cytogenetic studies are available on cases of ovarian hypoplasia/dysgenesis in alpacas and llamas, there are no reports on the histological features of this abnormality.

Anti-Mullerian Hormone (AMH), a member of the transforming growth factor-ß superfamily, is produced by the granulosa cells of the primordial follicles. AMH serum concentration has been used to predict the ovarian, primary follicular reserve in several species (9–11). In women, AMH serum concentration decreases significantly with the approach of menopause. Additionally, serum AMH levels are low in women with premature exhaustion of ovarian reserve (12, 13). AMH serum concentration has been used in dogs and cats to evaluate ovarian tissue (i.e., ovarian remnant syndrome) after ovariectomy (14, 15). In South American camelids, the role of AMH in reproduction remains poorly studied. Serum AMH assay has been validated in male alpacas pre- and post-castration (16). In the female alpacas, preliminary results show that serum AMH concentration could indicate ovarian reserve (17).

The objectives of the present study are to describe the clinical diagnosis and the histological features of ovarian hypoplasia/dysgenesis syndrome in alpacas. Additionally, we investigate whether ovarian dysgenesis correlates with low serum AMH levels. If so, the diagnosis of ovarian hypoplasia/dysgenesis could be made easier based on an evaluation of serum AMH.

## Materials and Methods

Five (5) female alpacas ranging from 18 months to 4 years of age, diagnosed clinically with ovarian hypoplasia by the Comparative Theriogenology service at the Veterinary Teaching Hospital, Washington State University (WSU) were included in this study. All females were maiden. The main complaint was continuous sexual receptivity and failure to become pregnant despite several matings to males with proven fertility. The females were donated by the owners and remained in the WSU teaching herd until euthanized for various reasons. All procedures conducted on the females were approved by the Institutional Animal Care and Use Committee.

### Clinical Examination

Females were subjected to a standard breeding soundness examination, including reproductive health history, physical examination, transrectal ultrasonography of the reproductive system, and vaginal examination. Transrectal ultrasonography was performed using a 7.5 MHz linear transducer mounted on a PVC handle at intervals of 3 days for 2 weeks as described by the authors (18). A vaginal examination was performed with a sigmoidoscope. Animals were suspected of lacking any ovarian follicular activity if the ovaries could not be visualized by ultrasonography. For confirmation of the diagnosis, females were examined by laparoscopy. Laparoscopic evaluation of the reproductive organs was performed as previously described (1, 19). Briefly, the animals were fasted for 24 h. Anesthesia was induced with a combination of ketamine (4 mg/kg, IM), xylazine (0.4 mg/kg IM), and butorphanol (0.4 mg/kg IM) then maintained with isoflurane in oxygen. The females were placed in dorsal recumbency, and the ventral abdomen was clipped and prepared for surgery. The examination was performed with a 10 mm rigid endoscope through a midline portal at the level of the umbilical scar after placing the animal in Trendelenburg position. A second portal was placed 10 cm lateral to *linea alba* to introduce grasping forceps to manipulate the viscera, and better visualize the reproductive tract. Post-operative care included antibiotics for 3 days and monitoring for any signs of discomfort. Upon confirmation of lack of ovarian activity, blood samples were obtained from the jugular vein in sodium-heparin tubes and submitted for cytogenetic evaluation at the cytogenetic laboratory at either the University of Minnesota or Texas A&M University.

### Euthanasia and Sampling and Histological Classification

Animals remained in the WSU teaching and research herd and were euthanized for various reasons. Humane euthanasia was achieved by intravenous administration of an overdose of sodium pentobarbital (40 mg/kg) (Euthasol®, Virbac AH, Inc., Fort Worth, TX). The reproductive tract was then harvested *in toto* for gross anatomic evaluation. The ovaries were excised and placed in 10% neutral buffered formalin. Paraffin-embedded sections were cut at 5 μm, stained with hematoxylin and eosin (H&E), and examined with an Olympus IX51 microscope equipped with TH4-100 digital camera. Blood samples were drawn from the jugular vein before euthanasia. Blood samples were allowed to clot then centrifuged to harvest serum which was stored at −80°C until analysis. Serum samples from reproductively normal females (*n* = 11) were used as a control for AMH serum concentration.

Histological classification of ovarian abnormalities as previously described by several authors as follows. Ovarian hypoplasia was defined as an ovary composed primarily of medullary connective tissue and blood vessels. The stroma is fibrous without generative elements or with only small number of primary follicles (8, 20, 21). Ovarian dysgenesis was defined as defective embryonic defective embryonic development of the gonad (streak gonads) (8). Gonadal dysplasia is defined as a gonad with architectural disorganization, with poorly defined morphologic boundary between the cortex and medulla irregular with a few primordial follicular structures (22).

### Anti-mullerian Hormone Assay

Serum AMH concentrations were determined in 50 μL duplicate aliquots of undiluted serum using a commercial enzyme-linked immunosorbent assay (Rat and Mouse AMH ELISA AL-113, Ansh Labs, Webster TX) as described by the manufacturer. According to the manufacturer, the antibody pair employed in this assay cross-reacts well with alpaca AMH. For validation, samples from alpacas, measured using a different commercial assay platform as validated and published previously, were re-analyzed using the Ansh (AL-113) platform. The concentrations of AMH in sera from five male alpacas, before and after castration, as measured using the Ansh assay, were highly correlated with the previously published results (*R*^2^ = 0.94, *n* = 10, and *P* < 0.01) (17).

Samples from each female were included in the same analytical run. Intra-assay coefficients of variation for serum pools with high (13.5 ng/mL, *n* = 14), medium (6.2 ng/mL, *n* = 14) and low concentrations (2.3 ng/mL, *n* = 14) were 2.6, 5.5, and 4.1%, respectively. The lowest calibrator was 0.41 ng/mL below which values were estimated by extrapolation.

Data on AMH level in alpacas with ovarian hypoplasia and control normal females were analyzed using a one-way ANOVA (Statistix 10®, Analytical Software, Tallahassee, FL).

### Karyotyping and Fluorescence *in situ* Hybridization (FISH)

Metaphase chromosome preparations were obtained from short term Concanavalin A (Sigma Aldrich) stimulated peripheral blood lymphocyte cultures following standard procedures (23). Chromosomes were G-banded (24) for identification, evaluated for count and morphology, and arranged into karyotype following the nomenclature proposed by Avila et al. (25). For each individual we will karyotype 5 cells. Animals with suspected minute chromosome were further studied by FISH using a probe specific for alpaca chromosome 36 and 18S-5.8S-28S ribosomal DNA (7) following a standard protocol (23).

## Results

On admission, all females were in gooden health with no history of previous illness. They were naturally mated by the owner starting at 15–18 months of age. All females displayed continuous receptivity despite multiple breedings with proven fertile males. Before referral, the number of matings (mean ± SD) was 11.8 ± 8.6 (range: 6–27). Reproductive evaluation by local veterinarians was limited to progesterone assay 2–3 weeks after mating. All samples had low progesterone level (<0.2 ng/mL).

**Figure 1:**
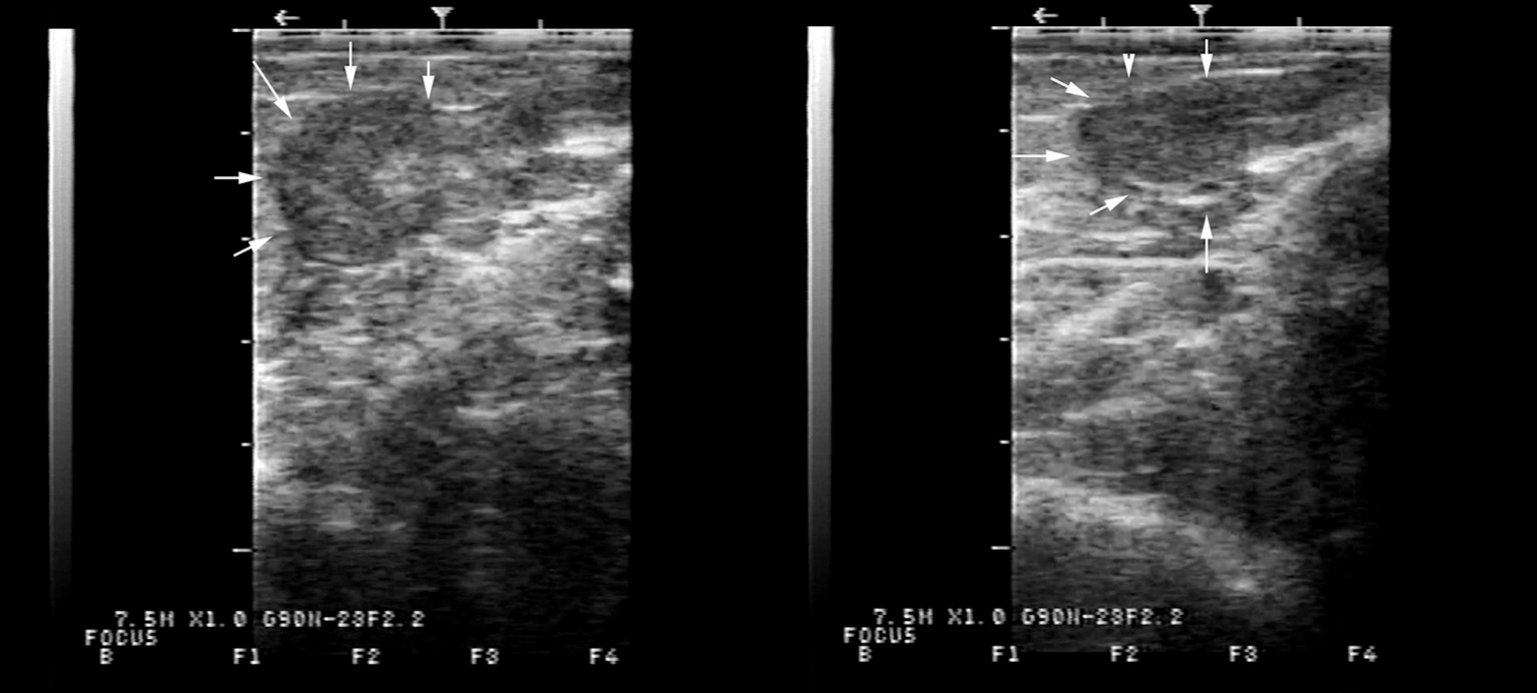
Typical appearance of the underdeveloped flaccid uterus on transrectal ultrasonography of two alpacas presenting with lack of ovarian activity. The uterus, delineated by arrows, measured in diameter, 6.5 mm in one case and 7.8 mm in the other.

**Figure 2:**
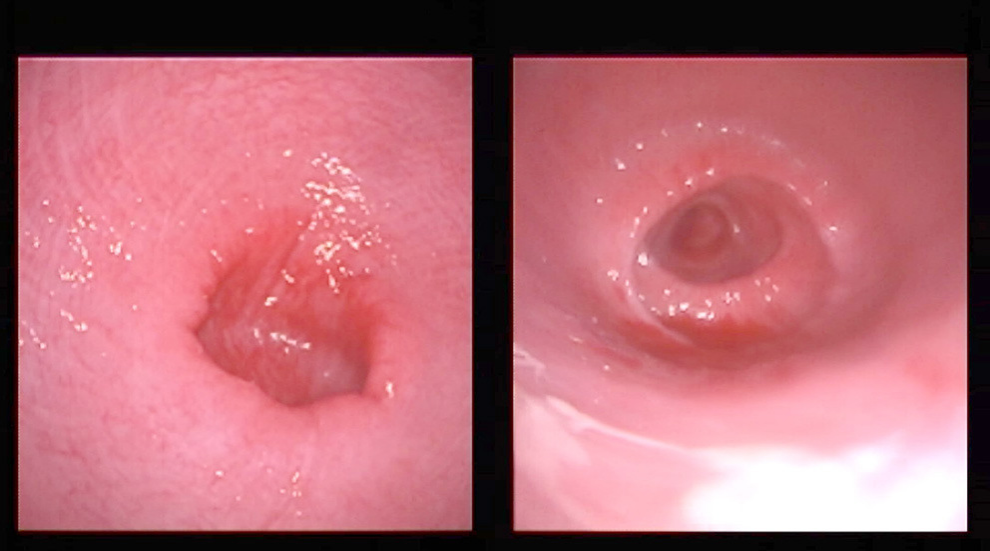
Typical appearance of the cervix by vaginoscopy from two females. The cervix is open and lacks tone.

On transrectal ultrasonography, all females presented a small flaccid uterus (Figure 1), and the ovaries could not be visualized. On vaginal examination, the most notable finding was a lack of tone in the cervix (Figure 2).

**Figure 3:**
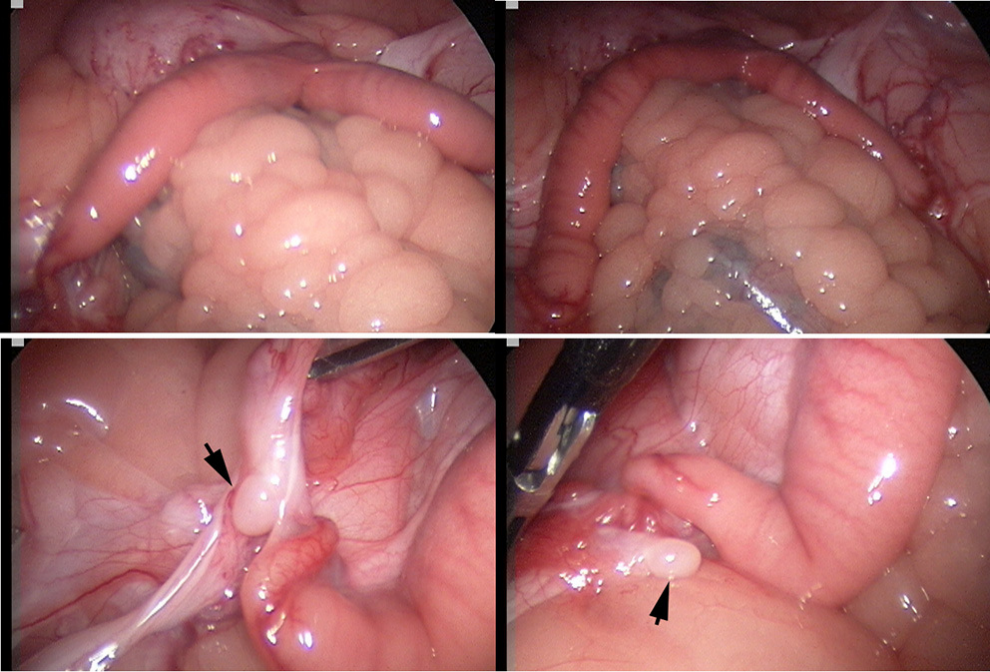
Laparoscopic evaluation of the reproductive tract: Top: infantile flaccid uterus, bottom: inactive small ovaries (arrows) with a smooth surface and no visible follicles.

**Figure 4:**
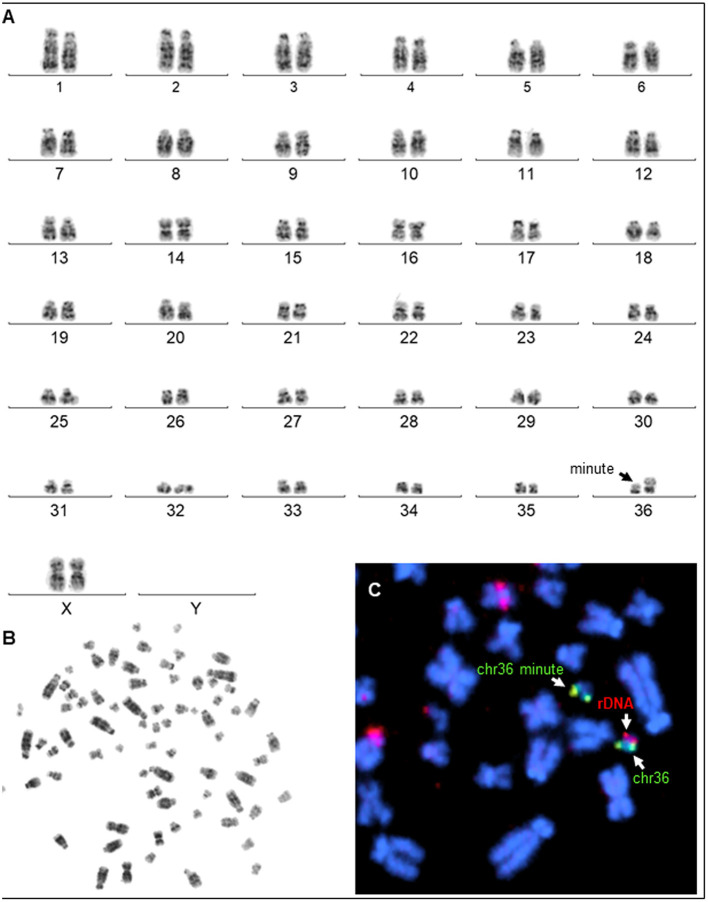
Karyotyping. **(A)** G-banded karyotype (74, XX, m) of a female alpaca with a minute chromosome (arrow showing size difference between homologs of chr36). **(B)** Corresponding metaphase spread. **(C)** Partial metaphase showing (arrows) FISH results with chr36 specific probe (green) and the presence of ectopic rDNA (red) in the larger chr36.

The laparoscopic evaluation confirmed the presence of a small flaccid uterus and extremely small ovaries (Figure 3). The cytogenetic evaluation revealed a normal female karyotype (74,XX) in five animals. However, one female presented a minute chromosome with clear size difference between chromosome 36 homologs (Figures 4A,B) due to ectopic presence of ribosomal DNA (rDNA) in the larger homolog (Figure 4C).

**Figure 5:**
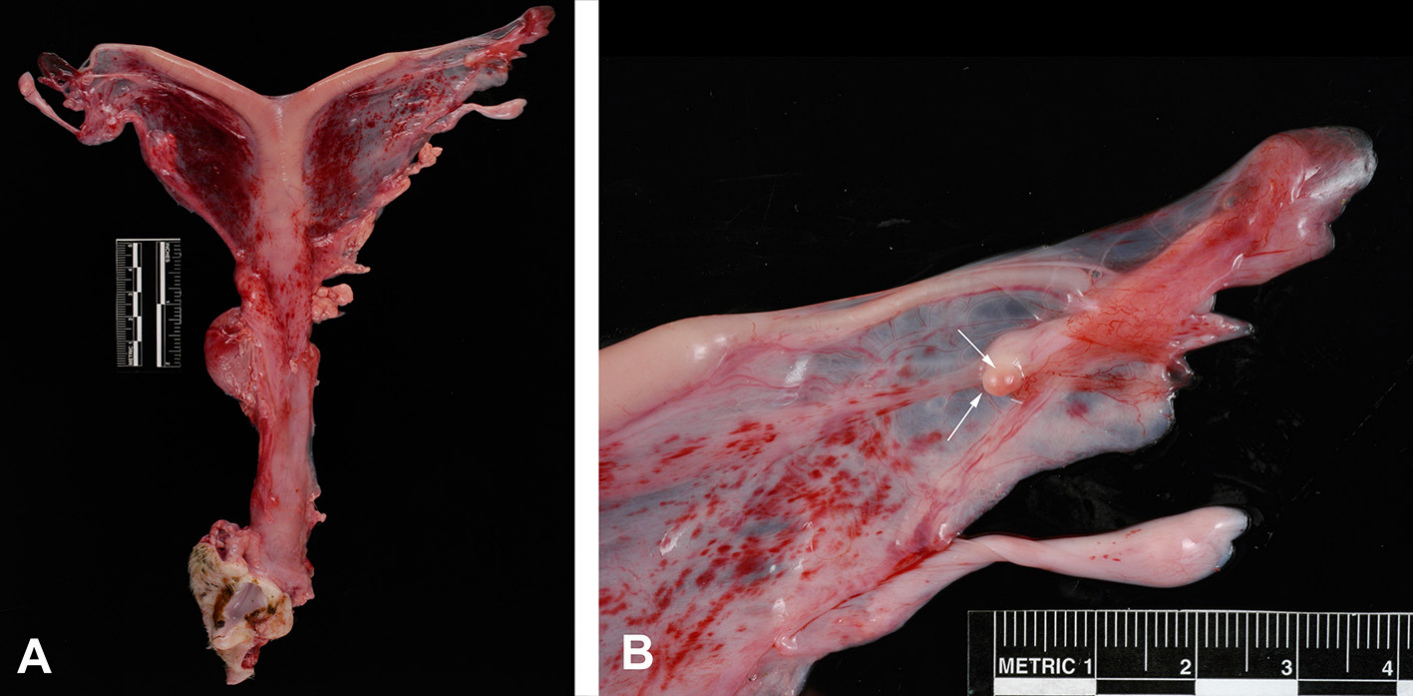
Reproductive tract from a female with ovarian hypoplasia/dysgenesis. **(A)** Entire tract showing the tubular genitalia with the small flaccid uterus. **(B)** Ovary measuring 3 mm in length by 2 mm in width (arrows).

**Figure 6:**
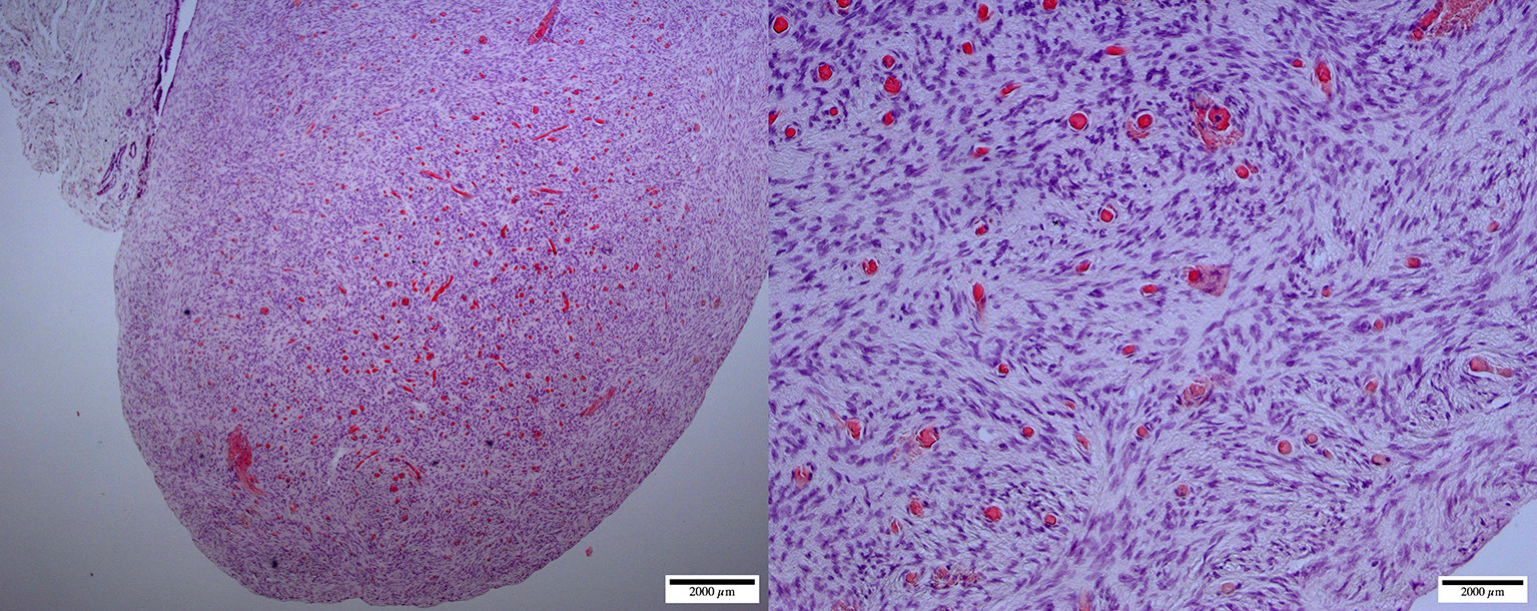
Ovarian hypoplasia (streak gonad): micrographs at lower (left, ×40) and higher (right, ×200) magnification of the ovary. Thick dense tissue with diffuse cortex and medulla containing a dispersed network of vascular structures. The cortex is devoid of any oocyte/follicular structures (H&E staining).

**Figure 7:**
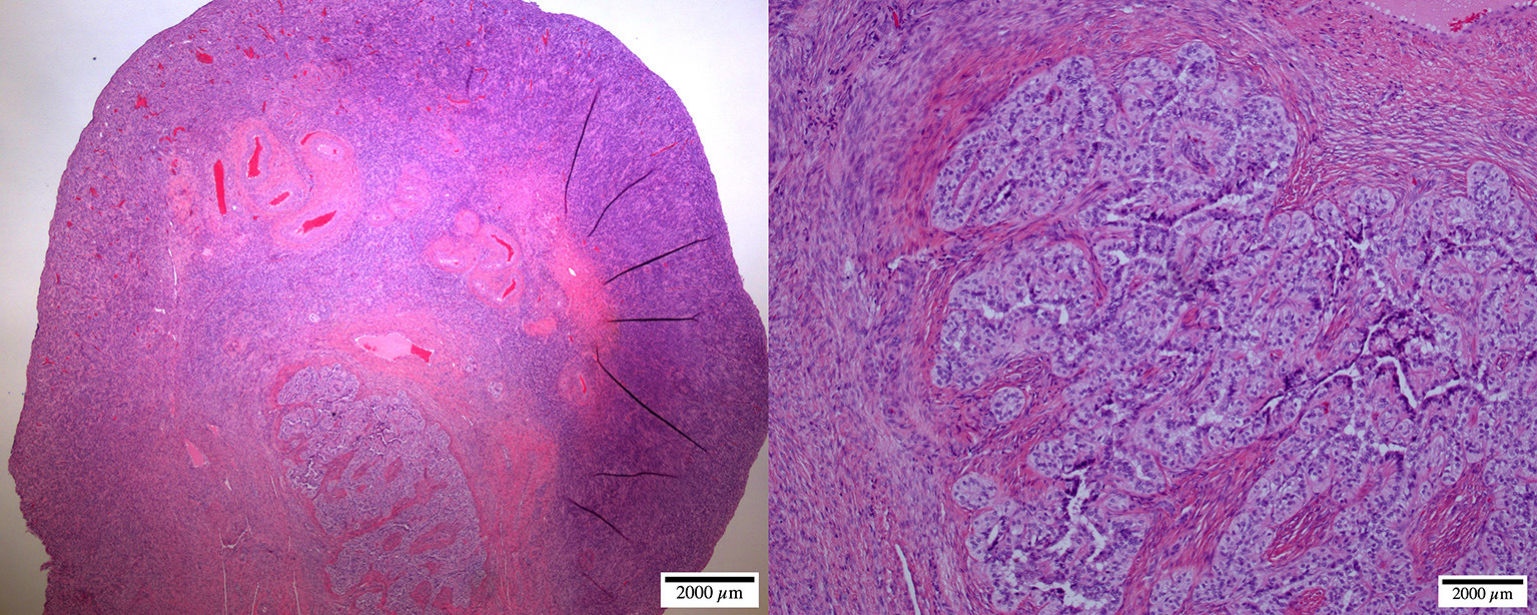
Ovarian dysgenesis: micrographs at lower (left, ×20) and higher (right, ×100) magnification of the ovary-like structure. The cortex is undifferentiated, compact, and devoid of any follicular activity. The medulla shows a network of undifferentiated ducts [reclassified as ovarian dysplasia according to (26)].

**Figure 8:**
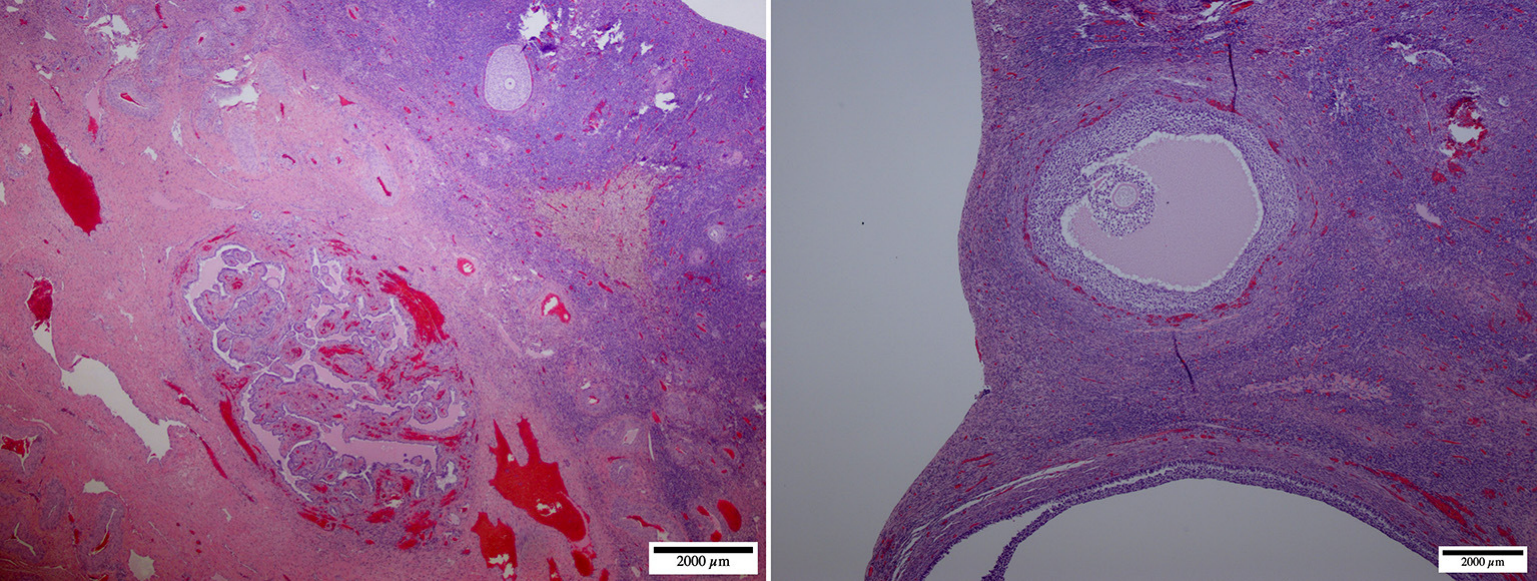
Ovarian dysplasia: micrographs at lower (left, ×20) and higher (right, ×40) magnification of the ovary. This case presented a poorly differentiated medulla. The cortex presented occasional primordial and primary follicles suggesting premature follicular reserve exhaustion [reclassified as premature ovarian insufficiency according to (26)].

Postmortem evaluation of the reproductive tract confirmed the presence of an infantile uterus and rudimentary ovaries (Figure 5). The left uterine horn's diameter (mean ± SD) was 6.4 ± 0.5 mm. The ovaries had a smooth surface with no visible follicular activity and measured between 3 and 4 mm (mean ± SD; 3.1 ± 0.3 mm). On histological evaluation, the ovaries from the five females could be categorized as either ovarian hypoplasia (*n* = 2; Figure 6), ovarian dysgenesis (*n* = 2, Figure 7), or ovarian dysplasia (*n* = 1, Figure 8).

**Figure 9:**
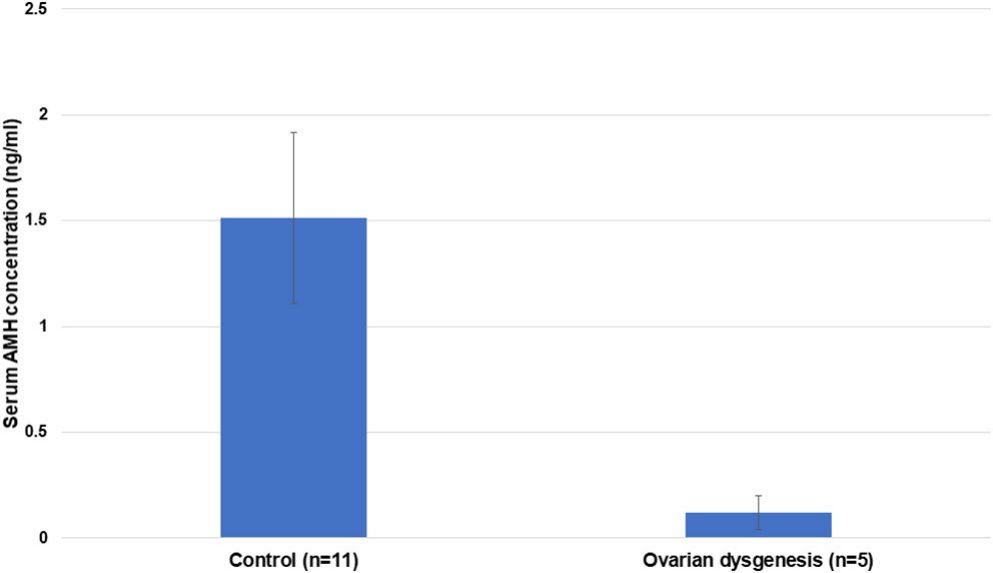
Mean ± SEM of serum AMH concentration in control (reproductively normal) (*n* = 11) and abnormal ovarian activity in females (*n* = 5) (*p* < 0.001).

Serum AMH concentration in affected females (mean ± SEM; 0.12 ± 0.08 ng/mL) was significantly lower (*p* < 0.05) compared to the control multiparous females (1.51 ± 0.4 ng/mL) (Figure 9).

## Discussion

Abnormal ovarian development is one of the most common congenital causes of infertility in several species of mammals (8). The definition of the syndrome is often confusing due to different histological presentations and classification. It may be described as ovarian hypoplasia (germ cell deficiency or low germ cell resistance), ovarian dysgenesis (i.e., defective embryonic development of the gonad), or ovarian dysplasia (abnormal follicular development). A human classification (26) was adapted to ovarian disorders in animals (http://vetrepropath.com, Dr. Rob Foster, University of Guelph, Canada) defines hypoplastic/streak gonad as the absence of tubules or follicles, and the ovary is largely composed of connective tissue and blood vessels; ovarian dysplasia is defined as an architectural disorganization with irregular, branched tubules and a fibrous interstitium; and premature ovarian insufficiency as a reduction of the follicular numbers or follicular reserve, and ovarian dysgenesis was excluded. Based on this classification the case in Figure 7 would be classified as ovarian dysplasia while the case in Figure 8 would be classified as premature ovarian insufficiency. Following this human classification, our cases would be categorized as either ovarian hypoplasia (*n* = 2; Figure 6), ovarian dysplasia (*n* = 2, Figure 7), or primary ovarian deficiency (*n* = 1, Figure 8).

Different types of gonadal hypoplasia have been reported in buffalo (27), cattle (20, 28–32), cats (33), dogs (33, 34), horses (35–37), and sheep (38).

Cytogenetic evaluation of these abnormalities has focused primarily on sex chromosomes. The partial or total absence of the X chromosome described in humans (Turner syndrome, Ullrich-Turner, Bonnevie-Ullrich-Turner) is one of the most common cytogenetic abnormalities (28). Gonadal hypoplasia/dysgenesis has been associated with X monosomy in several species, including alpacas, buffalos, cats, dogs, horses, llamas, and sheep (28). In alpacas and llamas, a peculiar form of chromosomal abnormalities referred to as minute chromosome syndrome (MCS) has been associated with ovarian hypoplasia (4, 6). The minute chromosome is characterized by the presence of an abnormally large homolog of chromosome 36 (chr36), so that the normal chr36 looks very small, thus the name *minute* (7) (Figure 4A). Size difference between chr36 homologs is due to heterologous and ectopic presence of nucleolus organizer region or rDNA (NOR) on the larger homolog (Figure 4C). It is theorized that such genetic difference between chr36 homologs may affect pairing, recombination, and segregation in meiosis (7). However, the molecular basis for this abnormality is still poorly understood (39). Only one alpaca was diagnosed with the MCS in the present set of clinical cases. The ovaries of this alpaca presented typical dysgenesis on histological evaluation. The remaining four patients did not show any cytogenetic abnormality. This suggests other, as yet unknown molecular mechanisms are involved in ovarian hypoplasia/dysgenesis in alpacas.

Gonadal hypoplasia is due to a failure of migration and synchronous mitotic division of primordial germ cells (PGCs) (40). In cattle, gonadal hypoplasia was found to be related to impaired migration of primordial germ cells. This disorder is frequent in two Scandinavian breeds of cattle, Northern Finncattle and Swedish Mountain cattle, and inherited in an autosomal recessive fashion with incomplete penetrance (32). This disorder was linked to an ectopic *KIT* copy number variation (32). The *KIT* gene encodes the tyrosine kinase family's type II receptor protein. KIT protein is crucial for survival proliferation and migration of the melanocytes precursors of PGC during embryogenesis (41). This type of gonadal hypoplasia in Scandinavian breeds of cattle was found to be correlated with the white coat color. Our observation in alpacas with gonadal hypoplasia/dysgenesis did not show any clear association with color. The cases presented in the study had variable coat coloration (black *n* = 1, white *n* = 2, and gray *n* = 2). In horses, ovarian dysgenesis is often associated with shorter stature which is another component of the X-monosomy phenotype. Although we have not taken direct measurements of the alpacas presented in this study, most of them were either of normal size or taller than normal.

As expected, serum AMH levels in alpacas diagnosed with ovarian hypoplasia/dysgenesis are lower than those found in control reproductively normal animals. Four out of five females in this study had undetectable serum AMH concentration, and the fifth had a very low level. This indicates that evaluation of a single serum sample for AMH could be used as a diagnostic tool for ovarian hypoplasia/dysgenesis. This is extremely important as in several cases referred to our theriogenology service, the condition has not been diagnosed due to a lack of clinical skills leading to frequent mating that jeopardizes the welfare of the animals.

## Conclusion

In conclusion, this study on ovarian hypoplasia/dysgenesis in alpacas revealed that the syndrome presents with several histological variants. The abnormality is not always associated with an abnormal karyotype, and that serum AMH concentration may serve as a method for definitive diagnosis without surgical intervention. However, more studies are needed to determine the etiology and heritability of this disorder in south American camelids. Additionally, there is a need for a better and more uniform histological classification of various ovarian development abnormalities.

## Data Availability Statement

The original contributions presented in the study are included in the article/supplementary material, further inquiries can be directed to the corresponding author/s.

## Ethics Statement

The animal study was reviewed and approved by Institutional Animal Care and Use Committee, Washington State University.

## Author Contributions

AT, EA, CP, and MC contributed to the conception and design of the study. AT wrote the first draft. TR contributed cytogenetic analysis and wrote section on cytogenetics. AC contributed hormone analysis and wrote section in material methods. All authors contributed to manuscript revision, read, and approved the submitted version.

## Funding

This study was supported by the Northwest Camelid Foundation Grant 13K-2530-0463, http://nwcamelidfoundation.org/.

## Conflict of Interest

The authors declare that the research was conducted in the absence of any commercial or financial relationships that could be construed as a potential conflict of interest.

## Publisher's Note

All claims expressed in this article are solely those of the authors and do not necessarily represent those of their affiliated organizations, or those of the publisher, the editors and the reviewers. Any product that may be evaluated in this article, or claim that may be made by its manufacturer, is not guaranteed or endorsed by the publisher.
